# Intranasal drug delivery: Unlocking the nose-to-brain route for central nervous system therapies

**DOI:** 10.1016/j.ijpx.2026.100521

**Published:** 2026-03-19

**Authors:** Ban Alwali, Thaigarajan Parumasivam, Moawia M. Al-Tabakha

**Affiliations:** a School of Pharmaceutical Sciences, Universiti Sains Malaysia, Penang, Malaysia; b College of Pharmacy and Health Sciences, Ajman University, Ajman, United Arab Emirates

**Keywords:** Intranasal drug delivery, Nose-to-brain drug delivery, Blood-brain barrier, CNS therapeutics, Intranasal devices, Nasal formulations

## Abstract

Intranasal drug delivery is rapidly gaining momentum as a game-changing approach for central nervous system (CNS) therapies, owing to its ability to bypass the blood-brain barrier (BBB) *via* olfactory and trigeminal pathways. It also enables systemic drug absorption while avoiding the metabolic drawbacks of oral administration, such as hepatic first-pass degradation. Recent advancements in intranasal formulation technologies, including *in situ* gels, nanoparticle-based systems, and permeation enhancers, have significantly enhanced drug retention, bioavailability, and targeted delivery. In addition, next-generation intranasal drug delivery devices, including bidirectional nasal sprays and the precision olfactory delivery (POD) technology, have further optimised drug deposition efficiency and enhanced therapeutic outcomes. Despite these advancements, several challenges persist, especially rapid mucociliary clearance in the nasal cavity and poor patient adherence, which limit clinical usage of intranasal medications. This review provides a comprehensive analysis of the anatomical considerations in drug absorption, formulation strategies, and technological advancements in intranasal drug delivery devices, emphasising its potential in overcoming traditional drug delivery barriers and unlocking new therapeutic potential for CNS and systemic applications. Future research should prioritise the enhancement of mucoadhesive properties, the optimisation of permeation enhancers, and the development of novel intranasal delivery devices to maximise clinical efficacy.

## Graphical abstract

**Figure:**
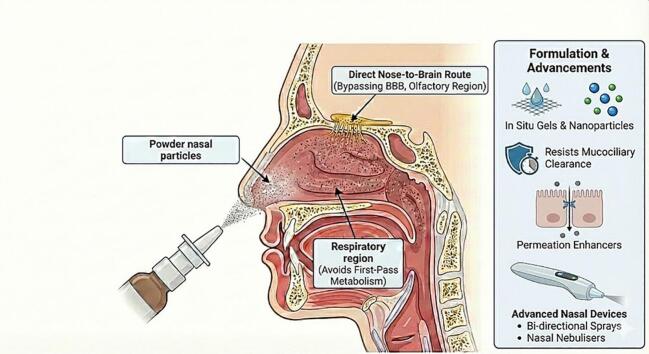

## 1 Introduction

Intranasal drug delivery has gained significant attention as a non-invasive route for both local and systemic therapies, particularly for treatments targeting the central nervous system (CNS). The nasal cavity is anatomically proximate to the brain, thereby allowing drugs deposited in the upper posterior regions to access the CNS *via* olfactory and trigeminal pathways as well as reducing reliance on transport across the blood-brain barrier (BBB). The intranasal route of administration also bypasses the gastrointestinal tract and hepatic first-pass metabolism. This is particularly relevant for peptides, proteins, and polar drugs that exhibit poor gastrointestinal stability, limited absorption, and low bioavailability (Luo et al., 2024).

The nasal cavity is highly vascularised and has a relatively large surface area, which can facilitate rapid systemic absorption of therapeutic agents. In adults, the nasal mucosa provides an absorptive surface area of approximately 160 cm^2^, which, together with high perfusion, may contribute to relatively rapid systemic drug absorption compared with many other non-parenteral mucosal administration routes (Chung et al., 2023). This facilitates a rapid onset of action and eliminates the necessity for painful injections or sterile preparations, positioning it as a versatile and efficient option for therapeutic interventions. This characteristic is crucial for improving patient compliance, as the absence of immediate symptomatic relief may prompt individuals to prematurely discontinue medication before achieving the desired therapeutic effect. Another advantage of intranasal drug delivery is its potential to complement other medications, whether administered orally or intravenously, particularly for elderly patients who may be managing multiple medications, thereby reducing incompatibility issues (Chung et al., 2023; Djupesland, 2013).

Despite its advantages, intranasal drug delivery has several disadvantages. Drug deposition can irritate or even damage the delicate nasal mucosa, particularly with frequent use, preservatives, or permeation enhancers, and improper device contact can traumatise the septum (Chung et al., 2023; Djupesland, 2013; Keller et al., 2022). Because only small volumes can be administered intranasally (approximately 100–150 μL per nostril), achieving therapeutic doses often requires highly concentrated formulations and therefore favours potent drugs (Chung et al., 2023; Madden et al., 2023). Incorrect administration technique leads to off-target deposition and mechanical loss (runoff or swallowing), reducing effectiveness (Chung et al., 2023; Djupesland, 2013; Al-Taie, 2025). In addition, rapid mucociliary clearance renews the mucus layer roughly every 10–20 min, limiting residence time and absorption (Chung et al., 2023; Keller et al., 2022). These constraints often demand formulation strategies that increase local retention and permeation while maintaining nasal tolerability with frequent dosing. Therefore, the successful design of an intranasal delivery system must holistically address these limiting factors.

This review synthesises current knowledge on (i) nasal anatomy and physiological determinants of absorption and deposition, (ii) the formulation approaches for intranasal drug delivery systems, and (iii) device technologies designed to improve targeting, particularly toward the olfactory region for potential nose-to-brain delivery. By integrating these aspects, the review aims to provide a comprehensive understanding of how intranasal delivery can optimise therapeutic outcomes, especially for CNS therapies.

## 2 Anatomy and physiology of the nasal cavity

The nasal cavity supports respiration, air filtration, olfaction, and speech, and its mucosa provides the primary interface for intranasal drug deposition and absorption. In adults, the cavity volume is approximately 15 mL and the mucosal surface area is approximately 160 cm^2^ (Chung et al., 2023), largely expanded by the turbinate structures (Khunt and Misra, 2021; Beule, 2010).

**Fig. 1:**
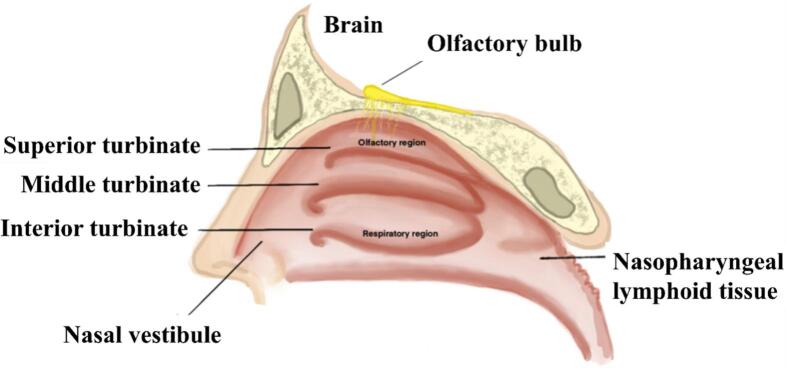
Schematic anatomy of the human nasal cavity (Created with BioRender).

Anatomically, the nasal cavity can be divided into the vestibule, respiratory region, olfactory region, and nasal-associated lymphoid tissue (NALT) located mainly in the nasopharynx (Fig. 1). The vestibular region is located in the anterior portion of the nasal cavity and encompasses an area of approximately 0.6 cm^2^ (Erdő et al., 2018). The region is characterised by a profusion of hair follicles, referred to as vibrissae, significant mucus production, and a robust squamous epithelial lining. Its primary role is to serve as a protective barrier by filtering inhaled air and trapping particulate matter, thereby defending against harmful environmental factors. These structural features, along with the limited permeability of the squamous epithelium, make intranasal absorption in this region minimal and pharmacologically insignificant (Erdő et al., 2018; Ibitoye et al., 2023).

The respiratory region is commonly referred to as the respiratory mucosa and includes the turbinates (conchae). It constitutes the largest section of the nasal cavity, measuring approximately 130 cm^2^ (Erdő et al., 2018). It is segmented into superior, middle, and inferior turbinates, which extend from the lateral wall. These specialised anatomical structures are essential for the humidification and thermoregulation of air inhaled into the respiratory system. Interposed between these structures are channels known as meatuses, which serve as passageways facilitating airflow and ensuring extensive interaction between the inhaled air and the respiratory mucosal lining. The nasal respiratory mucosa is characterised by a lining of pseudostratified, ciliated columnar epithelial cells and goblet cells, both of which play a critical role in intranasal drug delivery. Under normal physiological conditions, the nasal epithelium is coated with a thin layer of mucus secreted by glandular tissues and goblet cells (Khunt and Misra, 2021; Dahlstrom and Olinger, 2014). The nasal mucus layer measures merely 5 μm in thickness and is composed of two distinct sections: an outer layer that is thick and viscous, and an inner layer that exhibits a more liquid and serous consistency.

In terms of composition, this mucus layer is predominantly composed of water (95%), with the remaining constituents including mucin (2.5–3%) and a mixture of electrolytes, proteins, lipids, enzymes, antibodies, and bacterial by-products, which collectively account for approximately 2% (Beule, 2010; Leal et al., 2017).

The olfactory region is situated in the superior aspect of the nasal cavity and is characterised by a lining of specialised olfactory epithelium that encompasses olfactory sensory neurons (OSNs) and Bowman's glands (Chung et al., 2023). This neuroepithelium is unique in that it represents the only component of the CNS directly exposed to the external environment. This is a distinctive anatomical feature that allows intranasally administered drugs to bypass the BBB and reach the brain. As a result, the olfactory region serves as a valuable route for delivering CNS-targeted therapies, particularly in the treatment of neurological disorders such as Alzheimer's disease, Parkinson's disease, epilepsy, migraine, and multiple sclerosis (Jeong et al., 2023). While the olfactory epithelium is structurally similar to the respiratory epithelium in being pseudostratified, it contains specialised receptor cells essential for odour detection. Although it occupies only about 10% of the total surface area (2–12.5 cm^2^), its role in both olfaction and CNS drug delivery is critical (Sarma and Das, 2020).

On the other hand, the nasal cavity also hosts inductive immune tissues that make it an attractive site for vaccination. NALT, which functionally corresponds to Waldeyer's ring in humans (adenoids and tonsils), is a key site for immune surveillance. It contains organised lymphoid follicles with B- and T-cell areas, dendritic cells, and other antigen-presenting cells that efficiently sample intranasally delivered antigens (Kiyono and Fukuyama, 2004; Brandtzaeg, 2011). Intranasal immunisation can prime both mucosal and systemic immunity, typically inducing secretory IgA at mucosal surfaces and circulating IgG, thereby providing layered protection (Kiyono and Fukuyama, 2004; Neutra and Kozlowski, 2006).

From a pharmaceutical perspective, the anatomical configuration of the nasal cavity presents numerous advantages for drug delivery. For instance, the highly vascularised nasal mucosa facilitates a direct route for absorption into the systemic circulation, while the presence of turbinates increases the nasal mucosal surface area, supporting drug deposition and absorption. The nasal septum supports targeted local drug application, while the nasopharynx contributes to the onward transport of inhaled/deposited material (Jeong et al., 2023).

Although the anatomical attributes of the nasal cavity significantly amplify drug absorption and facilitate rapid therapeutic responses, the formulation characteristics of intranasal drug delivery are equally crucial for achieving optimal therapeutic outcomes (Illum, 2003; Pires et al., 2009). Essential physicochemical properties of the drug, including particle size and solubility, must be meticulously assessed to enhance intranasal absorption (Pardeshi and Belgamwar, 2013). Incorporating suitable excipients and carriers, including permeation enhancers, can further enhance the bioavailability of drugs administered *via* the intranasal route (Dhuria et al., 2010).

Formulation considerations also encompass the selection of appropriate delivery systems, which may include devices such as nasal sprays, nebulisers, and dry powder inhalers (DPIs), all of which are critical for the precise and controlled administration of pharmaceuticals (Arora et al., 2002). This ensures that the correct dose reaches the intended site of action while minimising the loss of drug particles. Thus, by effectively leveraging both the anatomical features of the nasal cavity and optimal formulations and drug delivery devices, it is feasible to maximise the therapeutic potential of intranasal drug delivery (Djupesland, 2013).

### 2.1 Physiological barriers to intranasal drug delivery

Despite the anatomical advantages of the nasal cavity, several physiological barriers can substantially limit intranasal bioavailability and targeting efficiency. The nasal mucosa is covered by a mucus layer. This viscoelastic barrier can hinder diffusion and trap particles, while coordinated ciliary beating transports mucus posteriorly (mucociliary clearance). The mucus layer thickness has been reported to be approximately 5 μm, and mucus transport velocities of approximately 5 mm min^−1^ that can rapidly remove deposited material from the nasal cavity (Modaresi and Shirani, 2023). Mucus renewal has been reported to occur within approximately 20 min, promoting loss *via* swallowing and anterior runoff when formulations do not sufficiently adhere or penetrate the mucus layer (Ulusoy et al., 2022). The clearance rates vary with age, disease state and environmental exposure (*e.g.*, tobacco smoke and air pollution) (Mall, 2008).

In addition to physical clearance, enzymatic and metabolic processes may reduce the fraction of intact drug available for absorption. The airway surface liquid and mucus contain proteases and peptidases (*e.g.*, aminopeptidases) that can degrade susceptible peptides and proteins, while the olfactory mucosa and olfactory bulb expresses xenobiotic-metabolising enzymes (including multiple CYP isoforms and phase II enzymes) (Minn et al., 2002). In addition, efflux transporters such as P-glycoprotein (MDR1) and members of the MRP family have been reported in nasal respiratory mucosa (Minn et al., 2002).

Nasal epithelial permeability also limits drug uptake, particularly for hydrophilic compounds and macromolecules. Transport across the nasal epithelium is strongly dependent on molecular size and lipophilicity. Small lipophilic molecules preferentially permeate *via* the transcellular route, whereas hydrophilic and high-molecular-weight compounds are largely restricted to paracellular transport through tight junctions, resulting in low absorption efficiency (Koch et al., 2023). These barriers necessitate formulations that are (i) prolong residence time, (ii) protect drugs from enzymatic degradation, and (iii) enhance permeability without compromising long-term nasal tolerability.

### 2.2 Mechanisms of nose-to-brain delivery

In direct nose-to-brain delivery, therapeutic agents may access the CNS primarily through the olfactory and trigeminal nerve pathways, thereby bypassing the BBB (Dhuria et al., 2010, Illum, 2000, Illum, 2003, Pardeshi and Belgamwar, 2013). The olfactory pathway is typically associated with delivery to the olfactory bulb and forebrain regions, whereas the trigeminal route is commonly linked to distribution patterns involving brainstem-associated structures (*e.g.*, pons/medullary regions) (Dhuria et al., 2010, Gänger and Schindowski, 2018, Illum, 2000, Pardeshi and Belgamwar, 2013, Pires et al., 2009). Trigeminal-mediated transport may be particularly relevant for drugs and formulations that predominantly deposit in the respiratory region (Jeong et al., 2023). The respiratory region, which forms the largest part of the nasal cavity, is highly vascularised, allowing drugs deposited there to be rapidly absorbed into the systemic circulation and potentially reach the brain after crossing the BBB (Jeong et al., 2023). In addition, the trigeminal nerve, which innervates both the respiratory and olfactory regions, provides a direct anatomical pathway from the nasal cavity to the brainstem and olfactory bulb (Johnson et al., 2010). Therefore, drugs that reach the trigeminal nerve will be transported directly to the brain while bypassing the BBB (Crowe et al., 2018, Drath et al., 2025).

On the other hand, drug transport may occur *via* extracellular mechanisms, such as bulk flow along perineural channels and paracellular diffusion through epithelial tight junctions, as well as intracellular mechanisms, including endocytosis into epithelial or neuronal cells followed by transcytosis and axonal transport (Arora et al., 2002, Dhuria et al., 2010, Illum, 2000, Pires et al., 2009). Generally, small and moderately lipophilic molecules may exhibit greater potential for transcellular diffusion across the nasal epithelium, whereas hydrophilic and high-molecular-weight therapeutics, such as peptides and proteins, often demonstrate limited passive permeability and may depend more heavily on paracellular transport and carrier-mediated uptake (Arora et al., 2002, Chung et al., 2023, Luo et al., 2024, Pires et al., 2009). Consequently, optimisation of nose-to-brain delivery is frequently achieved through a combination of targeted deposition in upper nasal regions and permeability-enhancing excipients as well as nanocarrier systems that protect the active pharmaceutical ingredient (API) from enzymatic degradation and facilitate mucosal transport (Chung et al., 2023, Dhuria et al., 2010, Illum, 2003). For example, low-molecular-weight, moderately lipophilic drugs may rely mainly on optimised deposition and solubilisation at the epithelial surface to support transcellular diffusion (Chaturvedi et al., 2011). In contrast, small hydrophilic drugs may benefit more from transient tight junction modulation and residence-time enhancement (Ozsoy et al., 2009). For macromolecules, passive diffusion is typically inadequate; therefore, protective delivery systems and uptake-promoting strategies, including carrier-based encapsulation, surface engineering to optimise mucus interaction and epithelial contact, and adsorptive or receptor-mediated transcytosis for appropriately designed systems, are commonly required to improve stability and facilitate intracellular transport processes (Chaturvedi et al., 2011, Chen et al., 2024).

At the cellular level, intracellular uptake in the nasal epithelium may involve multiple endocytic pathways, including clathrin-mediated endocytosis, caveolae/lipid raft-associated internalisation, macropinocytosis, and receptor- or adsorptive-mediated transcytosis, which can collectively influence the efficiency of CNS targeting (Dhuria et al., 2010, Pardeshi and Belgamwar, 2013). These pathways are particularly significant for macromolecules and surface-modified particulate systems, where ligand attachment or mucoadhesive coatings may enhance epithelial interaction and promote uptake, thereby increasing the likelihood of transport *via* olfactory and trigeminal neuronal pathways (Dhuria et al., 2010, Pardeshi and Belgamwar, 2013). Overall, efficient nose-to-brain transport depends on both extracellular and intracellular uptake mechanisms and on the design of formulations that align with drug properties (Chen et al., 2024, Gänger and Schindowski, 2018, Qiu et al., 2025).

## 3 Formulation considerations for intranasal delivery

When administering drugs intranasally, selecting the appropriate dosage form, whether liquid (solution or suspension) or dry powder, is crucial. Several formulation strategies, including dry powders, *in situ* gels, polymeric nanoparticles, and lipid nanocapsules, have been extensively investigated, as summarised in Table 1. The table highlights key findings on intranasal formulations for various diseases, outlining the formulation approaches and corresponding drugs. Generally, powder formulations offer greater stability than liquid forms. However, each form necessitates unique formulation strategies and presents specific optimal delivery and efficacy considerations. To determine the best formulation for intranasal drug delivery, several key factors must be evaluated to ensure effectiveness and patient comfort, including particle size, viscosity, pH, and volume of therapeutic agents, osmolarity, and the strategic incorporation of excipients (Scherließ, 2020).

**Table 1:** Summary of Nasal Formulations from Year 2019–2024 (This table summarises *in vitro*, *in vivo*, and clinical studies on nasal drug formulations published between 2019 and 2024).

Formulation	Drug	Excipient	Investigated Indication	Key Findings	References
Dry powder	Dexamethasone sodium phosphate	Mannitol or lactose monohydrate	Anti-inflammatory/olfactory delivery	Mannitol carriers improved flow/mucoadhesion and permeability; 3D nasal model showed ≈17% olfactory deposition.	(Nižić Nodilo et al., 2021)
*In situ* gel	Rizatriptan benzoate	Poloxamer 407, Carbopol 934P	Migraine	P407/Carbopol *in situ* gel showed adequate rheology and mucoadhesion with ≈8 h sustained release.	(Kantrodiya et al., 2019)
*In situ* gel	Zolmitriptan HCl	Sodium alginate, HPMC (K4M), chitosan HCl	Migraine	Mucoadhesive gel gave sustained release; chitosan enhanced permeability; formulation was stable.	(Malviya et al., 2020)
*In situ* gel	Paroxetine hydrochloride	Sodium alginate, HPMC, pectin.	Depression	*In situ* gel sustained drug release of ≈8 h, enhanced *ex vivo* permeation and improved antidepressant effect.	(Thakkar et al., 2021)
*In situ* gel (nano-lipid)	Teriflunomide	Glyceryl dibehenate, glyceryl monolinoleate, gellan gum, Carbopol 974P	Glioblastoma	Mucoadhesive gel doubled brain Cmax *vs* IN/IV NLCs in rats.	(Gadhave et al., 2021)
*In situ* gel (NLC-in-gel)	Resveratrol	NLCs (solid and liquid lipids), thermosensitive gel base	Neuroprotective/Alzheimer's	Thermosensitive NLC gel had suitable rheology/mucoadhesion and increased brain targeting and bioavailability *in vivo.*	(Rajput and Butani, 2019)
*In situ* gel (nanogel)	Paliperidone Palmitate	Poloxamer, guar gum	Psychotic disorders (schizophrenia)	Nanogel showed higher *ex vivo* nasal permeation compared to suspension; IN dosing increased brain exposure without EPS *in vivo*.	(Gadhave et al., 2023)
*In situ* gel (liposome-in-gel)	Sumatriptan Succinate	P407, Poloxamer 188, sodium alginate.	Migraine	Liposomes showed drug release of 86.11% over 8 h and increased brain exposure after IN dosing.	(Mathure et al., 2023)
Lipid nanocapsules (LNCs)	Mirtazapine	Labrafac MCT (oil), Solutol HS15 (surfactant), water	Depression	Optimised LNCs showed high brain targeting after IN dosing *in vivo*.	(Ibrahim et al., 2024)
Lipid nanocapsules (LNCs)	Nimodipine	Labrafac WL 1349 (MCT oil); Kolliphor (Solutol) HS15; Lipoid S100 (soy lecithin); NaCl	Subarachnoid hemorrhage	IN LNCs achieved brain levels comparable to IV with lower systemic exposure.	(Mohsen et al., 2020)
Liquid	Montelukast sodium	Hydroxypropyl cellulose (HPC), Carbomer 940 (C940)	Allergic rhinitis	Mucoadhesive spray (HPC/C940) gave sustained drug release; 0.01% C940 was non-toxic to nasal epithelium.	(Jullaphant et al., 2019)
Liquid and dry powder	Parathyroid hormone PTH (1–34)	Polyethylene Glycol (15)-Hydroxystearate (Solutol HS15)	Osteoporosis	IN bioavailability was ≈1% with rapid clearance in humans	(Pearson et al., 2019)
Liquid (spray)	Tranexamic acid	Hyaluronic acid (HA)	Post-operative healing/anti-inflammatory	> 90% nasal deposition; 0.3% HA reduced wound size (≈29%) and inflammatory markers; higher viscosity may prolong residence.	(Gomes dos Reis et al., 2020)
MSN-based spray	Fluticasone propionate (FP)	Mesoporous silica nanoparticles (MSNs) in aqueous spray vehicle	Allergic rhinitis/anti-inflammatory	FP-MSNs (≈400 nm) improved dissolution and were biocompatible/anti-inflammatory *in vitro*	(Mehmood et al., 2022)
Nanoliposome	Lamotrigine	Phospholipon 90G, cholesterol, Tween 80	Epilepsy	Nanosized liposomes had high entrapment and sustained release, enhanced *ex vivo* nasal permeation, and no nasal toxicity.	(Praveen et al., 2019)
Nanoparticles	Midazolam	Chitosan	Seizure emergencies	IN chitosan nanoparticles (≈147 nm) produced higher brain levels and brain:blood ratios compared to IV or IN *in vivo*.	(Shrestha et al., 2020)
Nanosuspension	Loratadine	Sodium hyaluronate	Seasonal allergies	Nanosuspension improved *in vitro* permeability and achieved ≈5.5 fold higher bioavailability compared to oral route in rats.	(Alshweiat et al., 2020)
Niosomes	Bromocriptine	Non-ionic surfactants, cholesterol, stabilisers	Parkinson's disease	IN niosomes showed sustained *in vitro* release; higher brain uptake and pharmacodynamic in rats; 28-day sub-acute IN toxicity showed no mortality or toxicologically significant changes	(Sita et al., 2020)
NLCs (chitosan-coated)	Donepezil HCl	NLC lipids matrix (solid+liquid, surfactants (stabilisers), chitosan coating	Alzheimer's disease	Chitosan-NLCs showed high EE, 24 h sustained drug release; greater *ex vivo* permeation, and higher brain exposure than IV *in vivo*.	(Yasir et al., 2022)
NLCs	Phenytoin sodium	Solid and liquid lipids.	Seizure control	< 50 nm NLCs released drug immediately, permeated olfactory epithelium best, and raised CSF/brain levels within 5 min the IV administration *in vivo*.	(Nair et al., 2021)
NLCs (in P407/Carbopol gel)	Aripiprazole	Stearic acid (solid lipid), castor oil (liquid lipid), Tween 80, P407, Carbopol 940	Schizophrenia	NLCs achieved ≈60% *ex vivo* permeation (*vs* ≈ 30% suspension), and showed no ciliotoxicity *ex vivo*.	(Albaqshi et al., 2024)
Polymeric particles	Ibuprofen	Chitosan-based thermosensitive hydrogel	Pain/inflammation	Hydrogel gelled at 30–35 °C, increased ibuprofen solubility and epithelial transport, and transiently opened tight junctions.	(Gholizadeh et al., 2019)
SLNs	Buspirone HCl	Glyceryl monostearate (matrix), Poloxamer 188 (surfactant).	Anxiety	SLNs (∼92 nm) sustained release 24 h, enhanced *ex vivo* permeation, and increased brain levels and anxiolytic effect.	(Yasir et al., 2021)
Solution	Fosphenytoin	HPMC, albumin.	Seizures/CNS delivery	IN formulations prolonged exposure with enhanced absolute bioavailability and lower Cmax.	(Pires et al., 2021)
Solution	Hydrocortisone	Biosurfactant (*Lactobacillus gasseri* BC9)	Corticosteroid therapy	BC9 biosurfactant increased solubility, showed mucoadhesion, and enhanced permeation across the *in vitro* models.	(Corazza et al., 2022)
Solution/spray	Rivastigmine	HPMC, Polysorbate 80; benzalkonium chloride.	Alzheimer's disease	IN solution favoured olfactory deposition and increased brain exposure.	(Guo et al., 2024)
Spanlastic vesicles (HPMC insert)	Lamotrigine	Span 60, Tween 80 (vesicles); HPMC (insert matrix)	Epilepsy	Vesicles loaded into HPMC inserts increased brain/plasma exposure after IN dosing *in vivo*.	(Abdelmonem et al., 2023)
Thermoresponsive lipid nanoemulsion	Temozolomide (TMZ)	P407 (gelation)	Glioblastoma (preclinical)	10% P407 nanoemulsion increased brain TMZ after IN dosing and reduced intracranial tumour *in vivo.*	(Michels et al., 2023)
Transniosomes	Naringin	Transniosomes	Epilepsy	IN transniosomes with suitable size, EE/ and stability raised brain naringin and produced significant antiepileptic effects *in vivo.*	(Gupta et al., 2024)

Abbreviations: IN, intranasal; IV, intravenous; CSF, cerebrospinal fluid; EE, entrapment efficiency; HCl, hydrochloride; MSN(s), mesoporous silica nanoparticle(s); NLC(s), nanostructured lipid carrier (s); SLN(s), solid lipis nanoparticle(s); HPMC, hydroxypropyl methylcellulose; P407, poloxamer 407; EPS, extrapyramidal symptoms; MCT, medium-chain triglyceride.

The development of formulations for intranasal drug delivery must also be guided by the physicochemical properties of the drug (Illum, 2003; Ugwoke et al., 2005). Critical factors include molecular weight (generally <1000 Da), lipophilicity (logP 1–5), aqueous solubility (> 100 mg/L), chemical stability in solution (preferably ≥2 years), and acceptable mucosal tolerability (Bitter et al., 2011). These factors collectively determine dose feasibility, epithelial permeability, and absorption efficiency. In addition, due to the limited administration volume within the nasal cavity, drugs intended for the nasal route are typically required to exhibit high pharmacological potency. These factors have been comprehensively reviewed in earlier reports (Bitter et al., 2011; Behl et al., 1998).

### 3.1 Particle size for deposition

The particle size of administered drugs significantly affects the efficacy of intranasal delivery. Research indicates that the optimal particle size for intranasal deposition ranges from 8 to 18 μm, though this may vary based on the delivery method and individual anatomical differences (Yarragudi et al., 2020; Ozmen et al., 2023). Particles with a volume mean diameter greater than 50 μm are primarily deposited in the anterior section of the nasal passage, while smaller particles (< 10 μm) tend to deposit in the anterior region and, to a greater extent, in the middle and posterior nasal sections (Calmet et al., 2019).

Other factors influencing the deposition of particles include airflow rate, nasal cavity structure, and delivery device design (Bass et al., 2022; Farnoud et al., 2020). Customising particle size and delivery techniques can improve the precision of drug delivery to the olfactory region, thereby bypassing the BBB. Understanding these dynamics is essential for enhancing intranasal drug delivery systems and their overall effectiveness. In addition, Warnken et al. demonstrated that demographic factors, including gender, age, and ethnicity, influence deposition patterns, which are vital for designing precise nasal drug delivery systems (Warnken et al., 2018). Li et al. also emphasised the significance of the spray angle for optimal delivery, noting that maximum deposition occurs when the angle exceeds 60^o^ with a slight head tilt (Li et al., 2018). Similarly, Shrewsbury et al. found that both the spray angle and delivery technique notably impact deposition within the turbinate region, highlighting the importance of spray characteristics in effective drug delivery (Shrewsbury et al., 2022). Hence, these findings underscore that both biological and technical variables must be carefully optimised to achieve consistent and targeted intranasal drug delivery.

### 3.2 Viscosity of Formulations

Viscosity plays a multifaceted role in intranasal drug delivery systems. Increasing viscosity can reduce mucociliary clearance and enhance mucoadhesion, thereby prolonging residence time and supporting absorption (Huang et al., 2024; Veronesi et al., 2020; Safarov et al., 2024). Polymeric agents such as polyvinyl alcohol and methylcellulose are commonly used to increase viscosity and improve residence time and, when optimised, can also improve patient acceptability (Horváth et al., 2016; Sharpe et al., 2003; Cai et al., 2011). However, the effect of viscosity on nasal delivery systems is determined by a balance between optimal spray performance during administration and sufficient retention of the formulation within the nasal cavity after deposition (Gao et al., 2020; Maaz et al., 2021). During actuation and atomisation, increasing viscosity resists liquid breakup and is generally associated with larger droplet sizes, narrower plume angles, and less anterior spreading, which can alter intranasal deposition patterns (Gao et al., 2020; Maaz et al., 2021; Pu et al., 2014). After deposition, moderate increases in viscosity can reduce post-nasal drip and slow mucociliary clearance, thereby prolonging mucosal contact time (Huang et al., 2024; Veronesi et al., 2020; Safarov et al., 2024; Pu et al., 2014). For instance, intranasal delivery of risperidone, a second-generation antipsychotic, formulated with poloxamer 407 (a thermoresponsive polymer) and HPMC (a viscosity enhancer and mucoadhesive agent) resulted in approximately 5.4-fold higher brain AUC compared with the oral solution when evaluated in male Wistar albino rats (Suhagiya et al., 2023; Khalil et al., 2024; Singh et al., 2025; Hard et al., 2025). Hence, viscosity should be regulated within a range that preserves adequate sprayability while still enhancing retention at the mucosal surface (Gao et al., 2020; Maaz et al., 2021; Pu et al., 2014).

Thixotropy is a property of a gel or fluid whose viscosity decreases under shear stress and recovers upon rest, offering a promising strategy for intranasal formulations. This unique behaviour enables formulations to be easily atomised during spraying while subsequently regaining viscosity to adhere strongly to mucosal surfaces (Kundoor and Dalby, 2011). Many intranasal corticosteroid (INCS) sprays use this principle through structured aqueous suspensions containing polymers such as microcrystalline cellulose and sodium carboxymethylcellulose. Similarly, Sailer et al. reported that the thixotropic gel nasal spray AM-301 could prevent nasal run-off post-application through re-gelation (Becker et al., 2024, Sailer et al., 2023). In clinical studies, barrier-forming thixotropic nasal gels/sprays improved symptoms of seasonal allergic rhinitis compared with saline or no treatment, with good tolerability (Becker et al., 2024, Nehrig et al., 2023, Struß et al., 2020). Collectively, these data indicate that thixotropic behaviour limits post-spray run-off and prolongs nasal residence, supporting improved intranasal exposure and clinical symptom control (Gao et al., 2020; Maaz et al., 2021; Pu et al., 2014; Casettari and Illum, 2014).

Chitosan and its derivatives have also demonstrated significant advantages in nasal drug delivery by enhancing residence time through strong mucoadhesion while simultaneously promoting epithelial interaction and transient opening of tight junctions, thereby facilitating drug absorption without relying solely on high bulk viscosity (Casettari and Illum, 2014). On the other hand, cyclodextrins offer a complementary strategy, particularly for poorly soluble drugs, as they enhance aqueous solubilisation and, in some cases, improve permeability and bioavailability, thereby enabling formulation optimisation without a proportional increase in viscosity (Rassu et al., 2018). In addition, *in situ* gelling systems offer a useful strategy for balancing sprayability and retention, because they can be administered as relatively low-viscosity liquids and subsequently undergo viscosity enhancement after deposition within the nasal cavity (Casettari and Illum, 2014, Guo et al., 2024, Pu et al., 2014). Overall, these strategies show that viscosity can be effectively modulated to enhance drug absorption.

In the context of CNS-targeted intranasal delivery, viscosity optimisation is particularly important because it can influence not only overall residence time, but also retention within the upper nasal region, where deposition on or near the olfactory epithelium is desirable for nose-to-brain transport (Maaz et al., 2021; Wang et al., 2024a; Guo et al., 2024). In this setting, the objective is not simply to increase viscosity, but to achieve a rheological window that limits rapid clearance and post-nasal drip while preserving acceptable spray plume characteristics and regional deposition (Maaz et al., 2021; Pu et al., 2014; Wang et al., 2024a; Koo et al., 2024). Recent studies have combined formulation optimisation with 3D-printed nasal cavity models, nasal casts, and related *in vitro* or *ex vivo* tools to quantify how formulation properties affect regional deposition and brain delivery (Maaz et al., 2021; Wang et al., 2024a). For example, Guo et al. developed a rivastigmine nasal spray for Alzheimer's disease and used a 3D-printed nasal cast to quantify olfactory deposition; the formulation containing 1% (*w*/*v*) Avicel (RC-591) as a viscosity modifier showed high olfactory deposition and enhanced brain delivery (Guo et al., 2024). Likewise, Wang et al. demonstrated that a borneol-modified lipid nanoparticle nasal spray with a viscosity of 39.36 millipascal-seconds significantly increased the olfactory deposition fraction to 23.58%, showing a strong correlation with intracerebral drug delivery compared with formulations exhibiting either higher or lower viscosities (Wang et al., 2024a). These findings support the view that viscosity modulation can contribute to nose-to-brain transport not only by prolonging residence, but also by improving regional targeting toward the olfactory epithelium (Guo et al., 2024, Wang et al., 2024a).

Furthermore, nanoparticulate nasal sprays developed for CNS disorders illustrate how viscosity optimisation can be integrated with carrier design to enhance brain exposure. Lipid-based nanocarriers and related nanosystems have been widely investigated for intranasal CNS delivery because they can provide controlled rheology, enhanced mucosal interactions, payload protection, and prolonged release (Koo et al., 2024; Trevino et al., 2020). Therefore, viscosity optimisation in CNS-targeted intranasal formulations should be considered in conjunction with regional deposition behaviour, carrier composition, and effective targeting of olfactory and trigeminal (Maaz et al., 2021; Koo et al., 2024; Trevino et al., 2020).

### 3.3 Volume and pH of therapeutic agent

The nasal cavity limits the deliverable dose volume; therefore, the target dose should be achieved by concentration or multiple actuations, not by increasing per-actuation volume. For most adult applications, volumes around ∼100–150 μL per nostril are recommended to balance drug retention/deposition with minimal nasal irritation, and many pumps meter ∼50–100 μL per actuation (Chung et al., 2023, Trevino et al., 2020).

On the other hand, maintaining the pH of the formulations (especially liquid formulations) within the range of 4.5 to 6.5 ensures optimal drug permeation, minimises nasal irritation, and inhibits bacterial growth. pH also plays a pivotal role in the chemical stability of drugs. Many peptide- and protein-based drugs are pH-sensitive and prone to hydrolysis, deamidation, or oxidation outside their optimal pH range, which can compromise therapeutic efficacy (Illum, 2003; Ugwoke et al., 2005). Therefore, maintaining pH not only enhances patient tolerability but also preserves the molecular integrity and potency of the therapeutic agent.

Determining the optimal dosage for nose-to-brain delivery requires meticulous consideration of both anatomical and formulation-related factors (Chung et al., 2023; Illum, 2003; Dhuria et al., 2010). Due to the limited volume that can be administered *via* the intranasal route, dosing strategies typically prioritise highly potent APIs or concentrated formulations capable of achieving therapeutic levels within small administration volumes (Chung et al., 2023; Illum, 2003). Dose selection is also influenced by the intended site of action, as delivery to the olfactory region may require a lower dose than systemic delivery (Jeong et al., 2023; Dhuria et al., 2010).

In practice, optimal intranasal dosing is often established through a combination of *in vivo* pharmacokinetic studies, brain-to-plasma exposure ratios, and pharmacodynamic endpoints, rather than relying solely on systemic dose equivalence (Dhuria et al., 2010). Formulation strategies that extend nasal residence time or enhance epithelial transport may also facilitate dose reduction while preserving therapeutic efficacy (Illum, 2003; Ugwoke et al., 2005). Consequently, dose optimisation for nose-to-brain delivery involves a balance between achieving a therapeutic concentration at the target site with minimal nasal irritation (Chung et al., 2023; Illum, 2003).

### 3.4 Osmolarity

Osmolarity is a critical driver of intranasal absorption and tolerability. Intranasal formulations are generally adjusted to physiological isotonicity (∼270–330 mOsm/kg), commonly achieved with 0.9% NaCl (≈0.154 M), to reduce stinging and mucosal irritation (Martin et al., 2021, Tonog and Nguyen, 2025, U.S. Food and Drug Administration (FDA), 2002). Preclinical studies, such as those conducted in rats, have shown that certain hypertonic NaCl concentrations (*e.g.*, ∼0.462 M) can transiently increase paracellular permeability and enhance uptake (Park et al., 2022), but hypertonicity can also provoke mediator release and should be balanced against epithelial integrity and comfort. Overall, tonicity should be optimised on a case-by-case basis, considering drug properties and dosage form, while staying near isotonicity for chronic use (Hemalatha et al., 2022, Illum, 2003, Ugwoke et al., 2005).

## 4 Excipients

Excipients used in intranasal formulations are not merely inactive additives but essential components that determine the safety, stability, and efficacy of the final product (Costantino et al., 2007). Excipients commonly used in intranasal formulations include mucoadhesive agents, preservatives, and permeation enhancers. These excipients play distinct and complementary roles in overcoming anatomical and physiological barriers, ultimately enhancing the therapeutic performance of intranasal formulations (Gänger and Schindowski, 2018).

### 4.1 Mucoadhesive agents

Polymers, such as chitosan, hypromellose, carbopol, carboxymethylcellulose and polyacrylic acid, are extensively utilised as excipients in intranasal formulations. These agents primarily serve to prolong the contact time of the formulation in the nasal cavity, thereby enhancing absorption. For instance, Shah et al. demonstrated that a chitosan-based mucoadhesive microemulsion offered superior brain-targeting capabilities compared with polymeric nanoparticles due to enhanced diffusion through the nasal mucosa, resulting in higher brain concentrations and greater intranasal bioavailability, making it a promising non-invasive technique for treating brain disorders (Shah et al., 2021). Similarly, Kannavou et al. revealed that chitosan-coated nanoemulsions were effective in delivering BNN27 (a synthetic C-17-spirodehydroepiandrosterone analogue) to the brain *via* intranasal administration, as the mucoadhesive properties of the chitosan increased the drug retention in the nasal passage and facilitated efficient brain disposition of the neurosteroid compared with liposomes (Kannavou et al., 2023). Thus, the incorporation of mucoadhesive agents in the formulation enhances bioavailability and improves drug adhesion to mucus, thereby facilitating better absorption.

### 4.2 Preservatives

Preservatives are essential for maintaining microbial stability and extending the shelf life of intranasal drug formulations. Common agents are benzalkonium chloride and the paraben family (methyl, propyl, and butyl hydroxybenzoates). However, some preservatives, especially benzalkonium chloride, have been associated with irreversible ciliary damage and have raised safety concerns for chronic use in intranasal preparations (Jiao and Zhang, 2019). This is particularly alarming because the WHO classifies benzalkonium chloride as corrosive to the respiratory tract and warns that aspiration can result in chemical pneumonitis (Kheirkhah Rahimabadi et al., 2022; International Labour Organization, 2021). These findings underscore the importance of carefully selecting and dosing preservatives in intranasal drug delivery systems to balance efficacy with safety.

Alternatively, the pharmaceutical industry is increasingly moving toward preservative-free formulations for intranasal delivery systems. By eliminating preservatives, manufacturers aim to enhance patient comfort and minimise the risk of allergies while maintaining adequate stability. Consequently, these formulations often employ multi-dose dispensers with specialised closure systems designed to preserve sterility over the product's shelf life, typically lasting up to 28 days (Marx et al., 2019). While single-use units naturally alleviate contamination concerns, multi-dose devices require a more complex setup, such as closed-system pumps to prevent external air entry, filters to eliminate airborne pathogens, and Bag-on-Valve (BOV) technology that encases the product in a sealed pouch to ensure sterility preservation (Ehrick et al., 2013). However, these advancements may reduce portability and incur additional production costs.

In some formulations, the preservatives not only inhibit microbial growth but also influence the ciliary movement within the nasal passages (Gänger and Schindowski, 2018). For instance, preservatives like chlorobutanol and hydroxybenzoates can induce reversible ciliostatic effects, slowing ciliary beating and potentially prolonging drug residence time; they also provide antibacterial and antifungal properties (Kheirkhah Rahimabadi et al., 2022; Marx et al., 2019).

Essential oils have also been investigated as potential antimicrobial agents due to their broad-spectrum activity, natural origins, and generally favourable safety profile. For instance, Kheirkhah Rahimabadi et al. examined the use of *Eucalyptus globulus* essential oil as a natural preservative in fluticasone propionate nasal spray, both alone and in combination with synthetic benzalkonium chloride (Kheirkhah Rahimabadi et al., 2022). This dual-preservative system effectively reduced microbial levels and provided significant preservative effects over 28 days. This highlights the potential of combining natural and synthetic agents to achieve effective preservation in intranasal preparations.

### 4.3 Permeation enhancers

Achieving sufficient intranasal bioavailability is critical for medications intended for CNS delivery. In intranasal delivery targeting the olfactory mucosa, overcoming the mucus barrier is the first challenge: drugs must dissolve in or traverse the mucus layer before encountering enzymatic degradation or mucociliary clearance (Rassu et al., 2018).

Permeation enhancers are agents that increase the permeability of nasal epithelial cells or membranes, facilitating drug diffusion and enhancing bioavailability (Maher et al., 2019). These enhancers can be categorised into various classes, including surfactants (such as bile salts and non-ionic surfactants), fatty acids, alkylsaccharides, cyclodextrins, chelating agents, and cationic polymers (*e.g.*, chitosan and its derivatives) (Maher et al., 2019). Mechanistically, permeation enhancement is typically achieved through transient modulation of epithelial tight junctions to increase paracellular transport, alteration of membrane organisation to facilitate transcellular diffusion, and may also influence local mucus-drug interactions or enzymatic stability depending on excipient chemistry and exposure profile (Maher et al., 2019). For instance, cyclodextrins can enhance the absorption of lipophilic drugs by forming inclusion complexes that improve apparent solubility and drug availability at the epithelial surface (Maher et al., 2019; Zhang et al., 2022).

Beyond classical chemical enhancers, cell-penetrating peptides (CPPs) represent a mechanistically distinct strategy that can facilitate epithelial drug uptake (*e.g.*, adsorptive endocytosis/transcytosis), particularly for hydrophilic and high-molecular-weight therapeutics. However, CPP selection and concentration require careful optimisation to ensure local tolerability while achieving meaningful transport enhancement (Hong et al., 2025).

A significant limitation of permeation enhancers is the potential for local mucosal irritation, impaired ciliary function, and epithelial disruption, particularly when surfactant-type enhancers are utilised at elevated concentrations or with prolonged exposure (Maher et al., 2019; Hermens et al., 1990). These risks become especially relevant during repeated or long-term administration, which is often necessary for CNS-targeted therapies. Consequently, mitigation strategies typically involve using the lowest effective concentration, prioritising rapid and reversible enhancement, limiting exposure through appropriate dose design, and minimising enhancer dependence *via* formulation strategies. For instance, the intranasal delivery of SA55, a broad-spectrum anti-SARS-CoV-2 monoclonal antibody (mAb), has been evaluated in a Phase I clinical trial for safety and tolerability in healthy volunteers (Hu et al., 2024). The formulation contained excipients including histidine hydrochloride, arginine hydrochloride, histidine, sucrose, polysorbate 80, hydroxypropyl cellulose, glycerol, and benzalkonium chloride (0.005%) (ClinicalTrials.gov, 2023). Participants received either a single dose (1 or 2 mg) or multiple doses for 7 days (1 or 2 mg per dose, 3 or 6 doses per day). The incidence of adverse drug reactions (ADRs) was 16.67% in the single-dose groups and 4.17% in the multiple-dose groups, demonstrating the favourable safety and tolerability of SA55 nasal spray in healthy volunteers (Hu et al., 2024). Similarly, intranasal ketamine was prepared from a 100 mg/mL solution and administered using a mucosal atomisation device (MAD) that delivers 0.1 mL per spray. Doses of 10, 30, and 50 mg provided significant analgesic effects and were generally well tolerated in patients with intractable cancer-related pain. Among these, the 50 mg dose produced the greatest pain relief without causing major adverse effects (Singh et al., 2022). Thus, intranasal formulations should be carefully balanced with safety considerations and clinical success, rather than relying heavily on permeation enhancers to achieve effective, well-tolerated therapy (Qiu et al., 2025).

In addition to conventional chemical enhancers and peptides, nanocarriers have emerged as a specialised class of delivery systems that can enhance apparent permeation and enable targeted drug delivery (Peng et al., 2020). Nanoparticle-based systems may achieve this by increasing epithelial contact and modulating interactions with the mucus layer. Polymeric nanoparticles, such as poly(lactic-*co*-glycolic acid) (PLGA), have been shown to facilitate particle penetration through the mucosa (Gänger and Schindowski, 2018). Additionally, PEGylation can be applied to minimise hydrophobic and electrostatic interactions between particles and mucus by ensuring the particles retain their hydrophilic characteristics through consistent coating (Peng et al., 2020). Negatively charged nanoparticles and hydrophilic excipients generally demonstrate reduced interaction with mucus, whereas positively charged and hydrophobic agents tend to interact more strongly. Hence, the careful design of drug delivery systems that consider physicochemical properties and mucosal barriers is essential for achieving therapeutic efficacy (Ghadiri et al., 2019).

Solid lipid nanoparticles (SLNs), nanostructured lipid carriers (NLCs), liposomes, nanoemulsions and niosomes have also emerged as promising nanocarrier-based delivery systems for intranasal drug delivery. These systems can enhance drug solubility, protect sensitive molecules from enzymatic degradation, and facilitate controlled or sustained drug release within the nasal cavity (Gänger and Schindowski, 2018; Ghadiri et al., 2019). These nanocarriers could also improve transport across the olfactory and trigeminal pathways by prolonging nasal residence time, enhancing epithelial penetration and enabling targeted delivery to the upper nasal regions (Dhuria et al., 2010; Gänger and Schindowski, 2018). Although these nanocarriers have been shown to enhance drug bioavailability and facilitate site-specific delivery, the precise pathways governing nose-to-brain transport remain incompletely understood, and their effectiveness in humans has yet to be clearly established.

## 5 Intranasal delivery devices

Intranasal delivery devices can be categorised according to dosage form (*e.g.*, drops, sprays, nebulisers, gels, and powders) and therapeutic objective (local nasal therapy or systemic or nose-to-brain delivery). They are commonly used for nasal irrigation (*e.g.*, isotonic or hypertonic saline) and for administering local medications such as corticosteroids, antihistamines, and antibiotics (Varricchio et al., 2023). Conventional devices predominantly deposit formulations within the anterior and middle regions of the nasal cavity, while deposition in posterior–superior regions remains limited (Illum, 2003; Dhuria et al., 2010). To overcome this limitation, specialised device designs have been developed to enhance delivery to posterior–superior targets, including the upper nasal space and olfactory region. These approaches employ mechanisms such as controlled airflow (*e.g.*, breath-powered bidirectional flow) and optimised plume geometry or nozzle orientation to facilitate transport beyond the nasal valve and minimise anterior deposition losses (Dhuria et al., 2010; Gänger and Schindowski, 2018). Table 2 compares various intranasal devices with respect to their deposition behaviour, key advantages, major limitations and the clinical contexts in which each platform is most appropriate.

**Table 2:** Comparison of intranasal devices: deposition characteristics, advantages, limitations, and clinical applications.

Device	Deposition target	Key advantages	Key limitations	Clinical Applications	Reference
Nasal drops	Gravity-driven and can reach middle meatus/olfactory cleft with positioning	Very simple/low-cost, useful when sprays are not feasible and can be directed with head position	Poor dose precision, runoff/swallowing and highly position-dependent	Saline, moisturisation and topical therapies	(Djupesland, 2013; Milk et al., 2021; Jacobson et al., 2024)
Conventional metered-dose nasal spray pumps	Mostly anterior/middle cavity and limited upper nasal space unless optimised	Metered dosing, portable, familiar to patients and fast administration.	Technique-sensitive, deposition is often restricted by the nasal valve and is variable in targeting posterior/superior regions.	Allergic/non-allergic rhinitis and many local therapies and some systemic products where deep targeting is not essential.	(Djupesland, 2013, Kundoor and Dalby, 2011)
Nasal nebulisers	Fine aerosol, potentially broader distribution and some sinonasal penetration	May improve coverage and useful when coordination is difficult	Longer administration time, bulkier and potential dose wastage	Sinonasal conditions, supportive care (*e.g.*, saline) and for patients who struggle with spray technique	(Moffa et al., 2019; Manes et al., 2011)
Breath-powered/ exhalation delivery (bidirectional) systems	Enhanced posterior/superior deposition and reduced soft-palate leakage	Better delivery and can improve targeting to difficult regions with reduced drip	Requires patient cooperation/exhalation, and may be coupled with costly devices.	Chronic rhinosinusitis/nasal polyps and other cases where deeper/superior deposition is desired	(Djupesland, 2013; Djupesland et al., 2006; Djupesland et al., 2020; Leopold et al., 2019; Sindwani et al., 2019)
Upper nasal space/ ‘precision’ targeting system (*e.g.*, POD-type)	Designed to reach the upper nasal space more consistently	Improved targeting consistency to the upper nasal space and less dependent on spray angle	Limited product-device combinations and can be costly	Rapid systemic/CNS onset where upper nasal deposition is prioritised (*e.g.*, migraine)	(Martin et al., 2021; Cooper et al., 2022; Trenkel and Scherließ, 2021; Shrewsbury et al., 2019; Smith et al., 2021)
Powder-based nasal devices	Possibly can support upper-nasal targeting in some systems	Better solid-state stability and portable	Dose limits and possibly nasal irritation	High-potency drugs that require stability and portability, including selected acute therapies and targeted delivery	(Trenkel and Scherließ, 2021; Tiozzo Fasiolo et al., 2018; Tepper and Johnstone, 2018; Lipton et al., 2017; Cady et al., 2015; Palmer et al., 2018)

### 5.1 Nasal drops

Nasal drops are commonly used for the administration of medications to infants and for simple treatments, such as saline solutions aimed at alleviating nasal congestion. This method is regarded as a straightforward and convenient approach for delivering medication to the nasal cavity. Nasal drops have the advantage of effectively reaching narrower anatomical regions, such as the olfactory cleft and the middle meatus, using gravitational forces (Trabut et al., 2020). This capability facilitates a more extensive distribution of the medication throughout the nasal cavity, particularly in the posterior region where the mucociliary activity is more pronounced (Tandel et al., 2011). Commercial examples include Otrivin Nasal Drops, Decozal Nasal Drops, and Fess Little Noses Saline Nasal Drops.

Proper head alignment is crucial when administering nasal drops as it significantly impacts both efficacy and patient adherence (Milk et al., 2021). The existing literature indicates that the Kaiteki position is more effective and comfortable compared to alternative positions, such as the head-back position (Milk et al., 2021; Jacobson et al., 2024; Trabut et al., 2020; Tai et al., 2021). A comparative study examining the exhalation delivery system and nasal spray found that nasal drops administered in the Kaiteki position were more effective in reaching the olfactory cleft, achieving an average score of 2.038 out of 3, whereas the head-back position scored significantly lower with an average of 1.058 (Jacobson et al., 2024). The scoring was performed using a semi-quantitative staining scale following administration of a fluorescein dye solution and nasal endoscopy was used to capture the staining patterns in the olfactory cleft. Deposition was graded from 0 (no staining) to 3 (heavy staining) after each administration (Jacobson et al., 2024). This finding indicates that a substantial volume of solution reached the olfactory cleft. Overall, administering nasal drops in the Kaiteki position effectively optimises delivery to the olfactory cleft.

A significant limitation of nasal drops is their imprecision in dosing when compared to other delivery devices, making them less suitable for medications that require strict dose accuracy (Tandel et al., 2011; Tai et al., 2021). However, advancements in nasal drop products have improved the dispensing system, for example: (i) by using more uniform dropper-tip geometries or flow restrictors to produce more consistent drop formation, (ii) ergonomic squeeze bottles that enable easier one-handed use, and (iii) user-friendly nozzle designs that help patients position the tip more consistently. These refinements can improve dose consistency (within the inherent limitations of drop-based delivery) and user convenience, particularly in home use.

### 5.2 Nasal sprays

Nasal sprays are designed to administer medications directly into the nasal passages *via* the nostrils. Marketed intranasal sprays are primarily classified into three categories based on their therapeutic application: antihistamines, corticosteroids, and topical decongestants. These sprays work by releasing a fine mist into the nostrils using a hand-operated pump mechanism (Saalbach, 2023). The medication is available either as a solution or a suspension, stored in a dispenser equipped with a one-way pump system that produces a metered dose when actuated. Usually, the device, consisting of a chamber, piston, and actuator, ensures the release of consistent and precise doses.

Nasal sprays typically produce aerosols ranging from 50 to 100 μm in diameter and deliver doses of 25–200 μL per actuation (Moffa et al., 2019; Tai et al., 2021). Due to their cost-effectiveness and ease of use, they hold a prominent role in the intranasal drug delivery market and serve as a frontline treatment for many nasal disorders. Examples include fluticasone propionate (*e.g.*, Flonase) for allergic and non-allergic rhinitis; mometasone furoate (Nasonex) for allergic rhinitis, prophylaxis of seasonal allergic rhinitis, and nasal polyps; xylometazoline (Otrivin) as a short-term decongestant for nasal congestion due to colds/allergic rhinitis/sinusitis; and the azelastine-fluticasone combination (Dymista) for seasonal allergic rhinitis in patients aged ≥6 years.

Nasal sprays offer several advantages over nasal drops, particularly their ability to deliver consistent metered doses, ensuring accurate therapeutic administration and minimising the risk of overdose-related side effects. They provide full and even dispersion of liquid formulations that effectively cover the nasal cavity and reduce dripping and anterior leakage (Moffa et al., 2023). However, similar to nasal drops, the orientation of the head significantly affects the deposition and effectiveness of nasal sprays. A backward tilt of the head can enhance intranasal absorption and therapeutic efficacy (Gao et al., 2020).

**Fig. 2:**
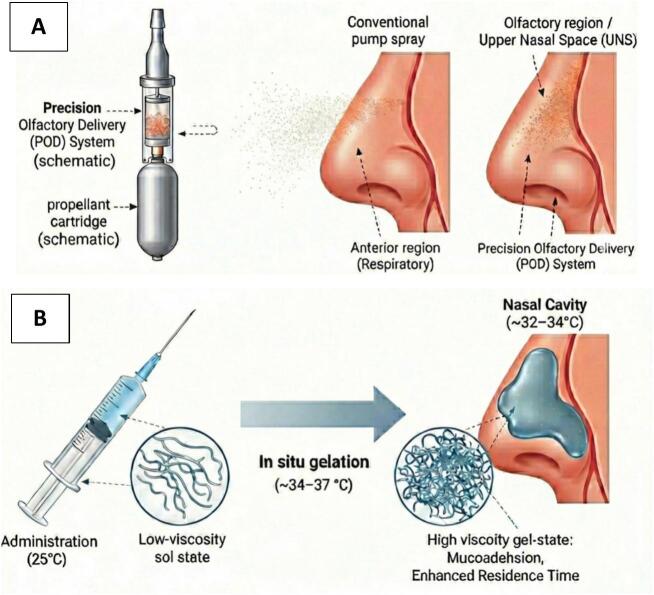
Schematic illustrations of (A) the upper-nasal precision (POD-type) targeting concept and (B) thermosensitive *in situ* nasal gelation. (A) Upper-nasal precision (POD-type) delivery concept compared with a conventional pump spray, designed to enhance deposition in the upper nasal space and olfactory region. (B) A low-viscosity sol is administered intranasally and undergoes *in situ* gelation under nasal conditions (*e.g.*, temperature and ionic environment), thereby increasing mucoadhesion and residence time.

Among these advanced platforms, the Precision Olfactory Delivery (POD) system is a handheld gas propellant device targeting the upper nasal space (UNS) (Cooper et al., 2022) (Fig. 2A). In a 99mTc-labelled peptide SPECT study in healthy volunteers, POD deposited approximately half of the intranasal dose in the UNS, markedly higher than a traditional pump, which placed most of its dose in the vestibule and < 5% in the UNS (Cooper et al., 2022; Shrewsbury, 2023). The device produces a biphasic emission, in which the propellant first launches droplets through the nasal valve and then deeper into the UNS, so deposition does not require breath coordination (Cooper et al., 2022). Together with clinical experience from POD-delivered dihydroergotamine (INP104) in migraine, these features support POD as a reliable, non-invasive platform with particular promise for neurological and psychiatric therapies (Starling et al., 2023).

### 5.3 Nasal nebulisers

Nasal nebulisers represent a promising alternative to conventional nasal sprays for intranasal drug delivery. Unlike nasal sprays, which often suffer from inconsistent deposition due to incorrect device orientation and patient technique, nasal nebulisers generate aerosol particles capable of penetrating the nasal cavity regardless of the device's positioning or inhalation angle. Clinical studies have also indicated that nasal nebulisation significantly enhances drug penetration into the sinuses, resulting in improved symptom relief and fewer systemic side effects when compared with oral or injectable alternatives (Manes et al., 2011). A notable example of a nasal nebuliser system is NasoNeb Nasal Nebuliser, an FDA-approved nasal device for delivering saline, nasal moisturiser, as well as over-the-counter and prescribed intranasal medications to the nasal cavities.

Nebulisers offer several advantages in terms of formulation compatibility (Hickey, 2020). Their ability to deliver aqueous formulations simplifies drug preparation and facilitates the administration of fragile biologics such as peptides, proteins, and other macromolecules that may degrade in dry powders or nasal sprays (Matthews et al., 2020; Forde et al., 2019; Longest et al., 2019). Moreover, nasal nebulisers have gained increasing popularity among patient populations unable to use nasal sprays or dry powder inhalers, particularly among young children aged four to five years. Despite these benefits, nebulisation has limitations such as longer administration time and increased medication waste, which may contribute to higher treatment costs (Longest et al., 2019).

### 5.4 Nasal gels

*In situ* gelling systems are advanced polymeric delivery formulations characterised by their ability to transition from a liquid to a gel state upon exposure to physiological triggers, such as temperature changes, pH fluctuations, or ionic interactions (Wang et al., 2013). For instance, thermosensitive polymers such as Poloxamer 407 remain liquid at room temperature but undergo micellisation and micellar packing to form a physically gelled network upon warming to nasal temperature (Wang et al., 2013; Qian et al., 2025). Conversely, ion-activated polymers such as gellan gum undergo rapid gelation upon interaction with endogenous cations (*e.g.*, Na^+^ and Ca^2+^) in nasal secretions, thereby promoting polymer chain association and junction-zone formation (Sherafudeen and Viswanadhan, 2015). Similarly, a pH-responsive gel at physiological pH alters polymer ionisation and swelling, increases viscosity, and forms a cohesive gel layer on the mucosa. Due to their ability to undergo a triggered phase transformation, they are often referred to as ‘smart materials’ (Qian et al., 2025). In intranasal drug delivery, these systems allow drugs to be administered as low-viscosity solutions for easy instillation and accurate dosing, transforming into a viscoelastic gel upon contact with the nasal mucosa (Fig. 2B). This transformation enhances mucosal adhesion, prolongs drug retention, and facilitates sustained drug release, ultimately improving bioavailability and therapeutic efficacy (Illum, 2000).

A significant advantage of *in situ* gels is their ability to mitigate mucociliary clearance, a key limitation of conventional aqueous nasal sprays or drops (solutions and suspensions) (Wang et al., 2024b). The effectiveness of these systems is largely influenced by their rheological properties, which provide adequate mechanical strength after gelation, mucoadhesion, and shear-thinning with rapid recovery for easy administration (Wang et al., 2024b; Wang et al., 2021). Several drugs have been formulated into *in situ* nasal gels, improving stability and delivery efficiency; for example, loratadine gels (HPMC/xanthan) showed sustained release (up to 6–10 h) and acceptable accelerated stability profiles, supporting their promise for allergic rhinitis (Sherafudeen and Viswanadhan, 2015). Similarly, mometasone furoate, a corticosteroid for allergic rhinitis, has been incorporated into a gellan-gum-based *in situ* gel (Cao et al., 2009). These formulations exhibit enhanced therapeutic efficacy due to increased residence time and improved drug delivery within the nasal cavity (Altuntaş and Yener, 2017). Commercial examples include NasoGel Spray, Ayr Saline Nasal Gel, and Rhinaris, which are moisturising OTC nasal gels indicated for dryness and soothing of the nasal passages.

Future research on *in situ* nasal gels is increasingly integrating nanotechnology and 3D printing. Nanoparticle-loaded gels are being explored to achieve targeted delivery, while 3D printing permits patient-specific formulation design with precise drug loading, geometry, and customised rheological properties. It can also incorporate multiple active agents or controlled-release layers within a single matrix, positioning *in situ* gels as a next-generation platform for advanced intranasal drug delivery (Zhang and Wang, 2022).

### 5.5 Powder-based nasal sprays

Nasal powder sprays are specifically designed for the insufflation of dry powder into the nasal cavity (Tiozzo Fasiolo et al., 2018). These devices typically accommodate unit-dose, bi-dose, or multi-dose systems, which consist of micron-sized active drug particles combined with appropriate powdered excipients. Intranasal powder formulations have been shown to be advantageous compared with liquid formulations because they provide improved stability, prolonged drug residence time on the nasal mucosa, and enhanced bioavailability (Tiozzo Fasiolo et al., 2018). This approach also eliminates the need for solubilising agents or propellants and reduces the degradation risks associated with liquid formulations. It is particularly effective for administering large molecules, such as peptides and proteins, which often exhibit poor stability in liquid formulations. Powder formulations also eliminate the need for cold chain storage, thereby enhancing their accessibility to developing countries. In addition, the absence of moisture minimises microbial growth, making the use of preservatives less necessary than in liquid formulations (Ehrick et al., 2013). Preservative-free powder formulations also allow for the administration of larger doses (Tiozzo Fasiolo et al., 2018). The maximum intranasal powder dose in humans is generally limited to approximately 50 mg per administration, depending on the powder's bulk density and dispersibility (De Barros et al., 2022). Excessive powder loads may cause patient discomfort, nasal dryness, irritation, and increased mucociliary clearance or sneezing, which can compromise dose deposition and reproducibility (Chung et al., 2023; Illum, 2003). Hence, intranasal powder formulations are more appropriate for highly potent APIs or for dosing regimens that divide the required daily dose into multiple administrations, rather than delivering a large single-dose payload (Chung et al., 2023; Illum, 2003).

One of the main drawbacks of powder-based intranasal administration is the tendency to cause nasal dryness or mild irritation due to particle deposition on the mucosal surface. Such effects are less common with liquid formulations, which, owing to their aqueous composition, are generally associated with lower irritation and better mucosal tolerability (Djupesland, 2013). Thus, continuous advancement in excipient design and device engineering will be essential to fully harness the therapeutic potential of nasal powder sprays while minimising their limitations (Keller et al., 2022).

Early powder formulations, such as Teijin's Rhinocort (beclomethasone dipropionate, Japan, 1986) and Erizas (dexamethasone cypionate, Nippon Shinyaku, Japan, 2012), were primarily designed for local treatment of hay fever (Trenkel and Scherließ, 2021). These formulations allowed for targeted drug delivery to the nasal mucosa, reducing systemic absorption and potential side effects. The first FDA-approved intranasal powder for systemic action, Onzetra Xsail (sumatriptan), was introduced in 2016 for migraine treatment, followed by Baqsimi (glucagon) in 2019, marking a significant shift toward systemic drug delivery *via* the intranasal route (Shrewsbury, 2023).

One notable advancement in intranasal delivery is the breath-powered Exhalation Delivery System (EDS), which uses patient exhalation to close the soft palate and generate positive pressure that propels drug beyond the nasal valve into the superior and posterior regions (Djupesland et al., 2020; Ow et al., 2023). EDS platforms exist for both liquid and powder formulations. For instance, XHANCE (fluticasone propionate delivered *via* EDS-FLU) was approved by the U.S. FDA in 2017 for nasal polyps and, in 2024, for chronic rhinosinusitis with and without nasal polyps in adults. Similarly, AVP-825 (Onzetra Xsail) is a sumatriptan nasal powder approved in 2016 for the acute treatment of migraine in adults. Collectively, EDS technology helps overcome anatomical barriers and has demonstrated clinical benefit in its approved indications (Ow et al., 2023).

## 6 Clinical translation and emerging therapeutic applications of intranasal drug delivery systems

In recent years, there has been a notable increase in the clinical translation of advanced intranasal delivery technologies, particularly those designed to overcome limitations associated with conventional nasal sprays, such as anterior deposition, post-nasal drip, and inconsistent dosing due to nasal valve obstruction. These innovations aim to enhance drug deposition and increase dosing consistency, both of which are particularly pertinent for therapies requiring rapid onset and improved intranasal targeting.

One example is the breath-powered intranasal powder delivery system AVP-825 (ONZETRA Xsail), which received U.S. FDA approval in January 2016 for the acute treatment of migraine in adults (Tepper and Johnstone, 2018). The device delivers a recommended dose of 22 mg sumatriptan nasal powder (11 mg per nostril) and has demonstrated improved therapeutic performance compared with oral administration in controlled studies. In the phase III trial, AVP-825 showed an earlier onset and consistent responses across multiple migraine attacks compared with 100 mg oral sumatriptan, supporting the clinical value of breath-driven delivery designed to improve deposition beyond the nasal valve (Lipton et al., 2017; Cady et al., 2015).

On the other hand, breath-powered bidirectional sprayers are engineered to address the nasal valve as a primary deposition barrier by utilising patient-generated exhalation to establish positive intranasal pressure and facilitate a “through-the-nose” airflow pathway (Djupesland et al., 2006). In the Exhalation Delivery System (EDS), exhalation through a mouthpiece closes the soft palate, directing the formulation from one nostril toward the superior and posterior regions of the nasal cavity before exiting through the contralateral nostril (Djupesland et al., 2020). This mechanism reduces post-nasal drip and the likelihood of pulmonary deposition while enhancing intranasal deposition beyond the anterior nasal passages. Comparative deposition studies have shown that EDS can improve delivery to target regions within complex sinonasal anatomies compared with traditional nasal sprays (Djupesland et al., 2006). Clinically, EDS technology has been incorporated into marketed therapies, including fluticasone delivered *via* EDS for chronic rhinosinusitis with nasal polyps, and has demonstrated significant treatment improvement in patients (Leopold et al., 2019). Despite these advantages, EDS may be less suitable for patients who are unable to generate reproducible exhalation manoeuvres and may entail greater device complexity and cost than conventional pump sprays.

Another recent platform is the Exhalation Delivery System (EDS), which employs positive-pressure exhalation to direct formulations deeper into the superior and posterior nasal regions while minimising pulmonary exposure and dosing variability (Palmer et al., 2018; Sher et al., 2020). The EDS has been integrated into XHANCE (EDS-FLU; fluticasone propionate nasal spray), which has been evaluated in clinical studies for chronic rhinosinusitis, including randomised controlled trials in patients with nasal polyps (NAVIGATE I and II) (Leopold et al., 2019; Sindwani et al., 2019). These trials demonstrated statistically significant and clinically meaningful improvements in symptoms and polyp outcomes with EDS-FLU compared with placebo (Leopold et al., 2019; Sindwani et al., 2019). In summary, these clinically approved platforms demonstrate successful translation of intranasal therapies beyond what can be achieved with conventional nasal sprays.

## 7 Author's opinion

Mucoadhesive agents play a critical role in intranasal drug delivery by counteracting rapid mucociliary clearance, thereby prolonging drug residence time at the administration site and significantly enhancing intranasal absorption and therapeutic efficacy. However, balancing adhesion strength with patient comfort presents a significant challenge. A primary concern is the limited toxicity data on commonly used excipients, particularly regarding their long-term effects on the nasal mucosa. To address this, regulatory bodies and researchers should prioritise developing standardised toxicity testing protocols for intranasal excipients, including chronic exposure models in animals and long-term follow-up in clinical settings.

Tidal breathing represents an underappreciated yet decisive factor in the success of intranasal drug delivery systems. Unlike forced inspiration, tidal breathing reflects the physiological state of patients during normal respiration, making it a more realistic determinant of intranasal drug deposition. Innovations like the NasoNeb system, which synchronises aerosol generation with natural breathing, highlight how engineering solutions can be guided by physiology to minimise drug loss and extend mucosal contact time. Hence, future formulation strategies must be designed with airflow dynamics in mind, rather than treated as independent variables.

It is clear that our understanding of the interactions among nasal mucosa, drug formulations, and delivery devices remains inadequate. The physiological and physicochemical factors that influence drug deposition, absorption, and clearance need further exploration. There is a need for computational models simulating intranasal deposition in different anatomical and pathological conditions, which could guide formulation optimisation and device design. In-depth studies are required to optimise formulation strategies and device designs, ensuring greater efficacy, safety, and patient compliance in intranasal delivery.

Future research should investigate the integration of emerging technologies to enhance intranasal drug delivery systems. For instance, 3D printing provides a novel platform for fabricating patient-specific nasal devices and dosage forms, thereby improving anatomical fit, comfort, and targeting efficiency. Approaches rooted in personalised medicine can further optimise formulation and dosing strategies by considering individual patient characteristics, such as mucosal properties and disease-specific factors. Furthermore, artificial intelligence (AI) and machine learning have the potential to facilitate predictive modelling of drug deposition, formulation behaviours, and therapeutic responses, thereby accelerating development and improving precision.

## 8 Conclusion

Intranasal drug delivery has emerged as a promising strategy for CNS therapies, offering rapid absorption and improved bioavailability through the olfactory and trigeminal pathways. Advances in formulation approaches, including *in situ* gels, nanoparticle-based systems, and permeation enhancers, have significantly enhanced nasal residence time and improved site-specific delivery. Nonetheless, challenges such as formulation stability, interpatient variability, and the need for user-friendly, reproducible devices remain unresolved. Future work should focus on defining quantitative design criteria rather than broad formulation modification. Specifically, studies should establish threshold values for viscosity and particle aerodynamic diameters required for deposition in the upper nasal region. Repeated-dose nasal safety studies incorporating functional endpoints (*e.g.*, mucociliary clearance rate and epithelial integrity) are also necessary. In parallel, device development should progress toward patient-adapted systems, including 3D printed devices tailored to individual nasal anatomy and airflow characteristics to maximise deposition in posterior–superior regions. Artificial intelligence (AI) and computational modelling are also expected to play a central role by enabling predictive simulation of airflow, particle trajectories, and regional deposition, as well as optimisation of formulation-device combinations and personalised dosing strategies. Finally, translational studies should focus on establishing robust *in vitro–in vivo* correlations that link regional deposition to brain exposure and pharmacodynamic outcomes, enabling rational dose selection and improved predictability across patient populations. Collectively, these advances will position intranasal administration as a non-invasive, effective, and patient-centred alternative to transform CNS therapeutics.

## Funding

The work was financially supported by Research University Team (RU Team) Grant from 10.13039/501100004595Universiti Sains Malaysia (Grant code: #2025/622/TE113).

## CRediT authorship contribution statement

**Ban Alwali:** Writing – original draft, Data curation, Conceptualization. **Thaigarajan Parumasivam:** Writing – review & editing, Supervision, Funding acquisition, Conceptualization. **Moawia M. Al-Tabakha:** Writing – review & editing, Supervision, Formal analysis.

## Declaration of competing interest

The authors declare that they have no known competing financial interests or personal relationships that could have appeared to influence the work reported in this paper.

## Data Availability

Data will be made available on request.
